# Non-anemic iron deficiency predicts prolonged hospitalisation following surgical aortic valve replacement: a single-centre retrospective study

**DOI:** 10.1186/s13019-022-01897-5

**Published:** 2022-06-16

**Authors:** Matthew C. Frise, David A. Holdsworth, Manraj S. Sandhu, Adrian J. Mellor, Adetayo S. Kasim, Helen C. Hancock, Rebecca H. Maier, Keith L. Dorrington, Peter A. Robbins, Enoch F. Akowuah

**Affiliations:** 1 Department of Physiology, Anatomy and Genetics, University of Oxford, Sherrington Building, Parks Road, Oxford, OX1 3PT UK; 2 Royal Berkshire NHS Foundation Trust, Royal Berkshire Hospital, London Road, Reading, RG1 5AN UK; 3 Oxford University Hospitals NHS Foundation Trust, John Radcliffe Hospital, Headley Way, Oxford, OX3 9DU UK; 4 Bristol Heart Institute, University Hospitals Bristol NHS Foundation Trust, Terrell Street, Bristol, BS2 8ED UK; 5 South Tees NHS Foundation Trust, The James Cook University Hospital, Middlesbrough, TS4 3BW UK; 6 Durham Research Methods Centre, NEDTC Hub, 1st Floor Arthur Holmes Building, Lower Mountjoy, South Road, Durham, DH1 3LE UK; 7 Faculty of Medical Sciences, Newcastle Clinical Trials Unit, Newcastle University, 1-2 Claremont Terrace, Newcastle Upon Tyne, NE2 4AE UK; 8 Academic Cardiovascular Unit, South Tees NHS Foundation Trust, The James Cook University Hospital, Middlesbrough, TS4 3BW UK; 9 Newcastle University, Newcastle Upon Tyne, NE1 7RU UK

**Keywords:** Cardiac surgical procedures, Anemia, Ferritin, Transferrin receptor, Aortic valve

## Abstract

**Background:**

Iron deficiency has deleterious effects in patients with cardiopulmonary disease, independent of anemia. Low ferritin has been associated with increased mortality in patients undergoing cardiac surgery, but modern indices of iron deficiency need to be explored in this population.

**Methods:**

We conducted a retrospective single-centre observational study of 250 adults in a UK academic tertiary hospital undergoing median sternotomy for non-emergent isolated aortic valve replacement. We characterised preoperative iron status using measurement of both plasma ferritin and soluble transferrin receptor (sTfR), and examined associations with clinical outcomes.

**Results:**

Measurement of plasma sTfR gave a prevalence of iron deficiency of 22%. Patients with non-anemic iron deficiency had clinically significant prolongation of total hospital stay (mean increase 2.2 days; 95% CI: 0.5–3.9; *P* = 0.011) and stay within the cardiac intensive care unit (mean increase 1.3 days; 95% CI: 0.1–2.5; *P* = 0.039). There were no deaths. Defining iron deficiency as a plasma ferritin < 100 µg/L identified 60% of patients as iron deficient and did not predict length of stay. No significant associations with transfusion requirements were evident using either definition of iron deficiency.

**Conclusions:**

These findings indicate that when defined using sTfR rather than ferritin, non-anemic iron deficiency predicts prolonged hospitalisation following surgical aortic valve replacement. Further studies are required to clarify the role of contemporary laboratory indices in the identification of preoperative iron deficiency in patients undergoing cardiac surgery. An interventional study of intravenous iron targeted at preoperative non-anemic iron deficiency is warranted.

## Background

Anemia is a well-established risk factor for adverse outcomes in patients undergoing cardiac surgery [1, 2] and in those with chronic heart failure (CHF) [3]. However, it is now recognised that iron deficiency (ID) is deleterious in CHF independent of anemia [4], and it has been demonstrated that intravenous (IV) iron supplementation in patients with CHF brings about substantial benefits not mediated by an effect on hemoglobin concentration [5–7]. Iron deficiency has a range of deleterious effects in cardiovascular disease (CVD) more generally [8], including acute coronary syndrome [9], idiopathic pulmonary arterial hypertension [10] and cyanotic congenital heart disease [11].

In a study in which bone marrow was sampled intraoperatively, 48% of patients of patients undergoing elective median sternotomy were found to have ID using bone marrow staining for iron [12], which is the gold-standard approach [13]. Remarkably, iron-deficient patients were more than twice as likely to have a normal preoperative hemoglobin concentration as they were to be anemic, highlighting that adequacy of body iron stores cannot be inferred from a normal preoperative hemoglobin concentration.

Studies exploring IV iron supplementation prior to cardiac surgery have typically focused on a transfusion-sparing effect [14–16]. Systematic reviews published in 2015 [17] and 2022 [18] have concluded that perioperative administration of IV iron in this setting is of uncertain benefit, whilst a recent observational study reported that IV iron therapy increased hemoglobin but had no effect on postoperative outcomes [19].

Iron deficiency in patients undergoing cardiac surgery is heterogeneous [20]. Various ID states with differing etiologies exist [13], including iron-deficiency anemia (IDA), non-anemic iron deficiency (NAID), inflammation-driven iron sequestration, and anemia of chronic disease. In some situations, IV iron might be helpful; in others, it might be ineffective or deleterious. A prerequisite for determining whether iron supplementation is beneficial is proper characterisation of ID in cardiac surgery patients including measurements of modern indices of iron homeostasis. Accordingly, we sought to characterise ID in a cohort undergoing surgical aortic valve replacement, from the previously published MAVRIC trial [21]. We hypothesised that ID would show different associations with postoperative outcomes in this setting depending on the index of iron status used to define it.

## Methods

This was a retrospective, observational, cohort study. The MAVRIC trial recruited individuals undergoing median sternotomy for isolated AVR [21]. The trial explored whether manubrium-limited ministernotomy reduced blood transfusion requirements. Patients presenting with features requiring that they undergo surgery prior to discharge were classified as ‘urgent’; those admitted for a planned procedure were classified as ‘elective’. We measured indices of iron status using stored plasma samples. The primary objective was to determine the prevalence of preoperative ID in patients undergoing surgical AVR. The secondary objective was to examine associations with transfusion requirements and length of cardiac intensive care unit (CICU) and hospital length of stay (LoS).

Iron deficiency was defined according to two different criteria. First, using plasma ferritin < 100 µg/L (ID_Fer_). Second, using plasma sTfR > 28.1 nmol/L (ID_sTfR_). The former was employed by an early study of IV iron supplementation in CHF [5], and has since been widely adopted in cardiopulmonary disease more generally [8]; the latter was derived from an early study of sTfR levels in healthy individuals [22]. sTfR has similarly been widely used in cardiopulmonary disease research [8]. Corresponding definitions for iron repletion (IR) were plasma ferritin ≥ 100 µg/L (IR_Fer_), and plasma sTfR ≤ 28.1 nmol/L (IR_sTfR_). Anemia was defined according to World Health Organisation guidelines as [Hb] < 130 g/L in men and [Hb] < 120 g/L in women [23].

Aliquots of EDTA plasma, obtained by centrifugation and stored at − 80 °C, were assayed for C-reactive protein (CRP), ferritin and transferrin at a University Hospital clinical pathology laboratory. Serum samples were unavailable, so determination of transferrin saturation (TSat) was not possible. Plasma sTfR (Quantikine®, R&D Systems, Abingdon, UK), and hepcidin (Hepcidin 25 HS, DRG, Marburg, Germany) were analysed by enzyme-linked immunosorbent assay.

Data were analysed by an independent statistician. The mean and standard deviation were determined, and for non-normally distributed variables, the median and interquartile range. Fisher’s exact test was used to compare prevalences of ID and anemia. Logistic regression analysis of associations of ID dichotomised according to sTfR and ferritin was undertaken, including with LoS. All comparisons were 2-sided taking *P* < 0.05 to be statistically significant. Additionally, the data were explored using descriptive statistics. Analyses were performed using R 3.3.3 (The R Foundation) and SAS 9.4 (SAS Institute Inc).

## Results

Preoperative blood samples were available for 250 patients, all of whom were included in the analysis. The mean (SD) age was 69.1 (8.9) years and 61% were male.

## Prevalence of iron deficiency

The prevalence of ID_Fer_ and ID_sTfR_ are shown in Table 1. The prevalence of ID was significantly higher using the ferritin-based definition compared with the sTfR-based definition (60% v. 22%, respectively; *P* < 0.0001, McNemar’s test). Most of this difference was accounted for by an excess of patients identified by a low plasma ferritin as having NAID.

**Table 1:** Prevalence of iron deficiency in the cohort

	Prevalence, *n* (%)		
Definition of ID used	ID	IDA	NAID
sTfR > 28.1 nmol/L (ID_sTfR_)	55 (22%)	23 (9.2%)	32 (12.8%)
Ferritin < 100 µg/L (ID_Fer_)	149 (60%)	36 (14.4%)	113 (45.2%)

*ID* Iron deficiency, *IDA* Iron-deficiency anemia, *NAID* Non-anemic iron deficiency

## Relationship between anemia and iron deficiency

Forty-seven patients (19%) were anemic. As shown in Table 2, the prevalence of ID was, as expected, significantly higher in anemic than non-anemic patients, irrespective of the definition used. Using sTfR to define ID identified 49% of anemic patients as iron deficient, but only 16% of non-anemic patients. Using ferritin to define ID identified 77% of anemic patients as iron deficient, as well as 56% of those who were non-anemic. Thus, ferritin identified the majority of both anemic and non-anemic patients as iron deficient.

**Table 2:** Prevalence of iron deficiency according to presence or absence of anemia

	Iron deficiency prevalence, *n* (%)	
Presence of anemia (*n*)	ID_sTfR_	ID_Fer_
Anemic (47)	23 (49%)	36 (77%)
Non-anemic (203)	32 (16%)	113 (56%)

The absolute difference in ID_sTfR_ prevalence between anemic and non-anemic patients was 33% (49% minus 16%; 95% CI: 18–48; *P* < 0.0001); for ID_Fer_ the value was 21% (77% minus 56%; 95% CI: 7.0–35; *P* = 0.0084). However, using the ID_Fer_ definition identified a greater proportion of non-anemic patients as iron deficient than did ID_sTfR_. The absolute difference in prevalence of ID in non-anemic patients using the different definitions was 40% (56% minus 16%; 95% CI: 31–49; P < 0.0001). Thus, sTfR gave a considerably more conservative estimate of the prevalence of NAID than did ferritin.

## Characteristics of patients according to iron status

Table 3 shows the characteristics of patients when split by iron status using sTfR or ferritin. Neither definition revealed a significant association of iron status with age or urgency of operation. Both gave significant differences in anemia prevalence, mean [Hb], transferrin, and hepcidin according to iron status; in each case the associations were similar: iron-deficient patients had lower [Hb], higher transferrin and lower hepcidin. However, the ID_Fer_ and ID_sTfR_ definitions differed with respect to sex and CRP. Using ferritin identified more female patients as iron deficient; an sTfR-based definition showed no such association with sex. Patients identified as IR_Fer_ had significantly elevated CRP levels–consistent with greater inflammation–whereas no difference was seen in CRP levels between ID and IR groups using iron status determined using sTfR.

**Table 3:** Characteristics of patients according to iron status

	Ferritin			sTfR		
Parameter	ID	IR	ID v IR mean difference (95% CI)	ID	IR	ID v IR mean difference (95% CI)
Age–y	70 (9.2)	68 (8.3)	2.1^NS^ (− 0.1﻿, 4.4)	70 (9.2)	69 (8.8)	0.6^NS^ (− 2.1﻿, 3.3)
Male–%	56	68	− 12.6* (− 24.7, − 0.5)	64	60	3.6^NS^ (− 10.8﻿, 8.1)
Urgent–%	13	19	− 6.1^NS^ (− 15.4, 3.3)	22	13	8.5^NS^ (− 20.4﻿, 3.4)
Anemic–%	24	11	13.3* (4.1﻿, 22.4)	42	12	29.5* (15.7﻿, 43.3)
Hb–g/L	134 (15)	143 (14)	− 9.2* (− 12.9, − 5.5)	132 (21)	140 (13)	− 7.9* (− 12.5, − 3.4)
Transferrin –g/L	2.7 (0.4)	2.4 (0.3)	0.3* (0.2, 0.4)	2.7 (0.4)	2.5 (0.4)	0.2* (0.1﻿, 0.3)
CRP–mg/L	3.6 (4.5)	9.6 (29.8)	− 6.1* (− 10.9, − 1.2)	7.2 (14)	5.7 (21)	1.4 ^NS^ (− 4.4﻿, 7.3)
Hepcidin –µg/L	21 (16)	59 (33)	-37.8* (− 43.9, − 31.5)	21 (22)	41 (32)	− 19.5* (− 28.4, − 10.5)
Ferritin–µg/L	–	–	–	79 (82)	120 (108)	− 41.5* (− 72.6, − 10.5)
sTfR–nmol/L	25 (9.5)	21 (6.7)	4.5* (2.3﻿, 6.6)	–	–	–

Values are given as the mean (SD). For comparisons between groups the mean difference and 95% confidence interval (CI) are given. Where the CI includes zero the difference is marked as NS (non-significant); where the CI does not include zero an asterisk indicates a statistically significant difference

## Red blood cell transfusion requirements

As shown in Table 4, iron status, however defined, was not associated with increased risk of receiving one or more units of red blood cells.

**Table 4:** Incidence of perioperative red blood cell transfusion

		Patients transfused at least one unit of red cells, *n* (%)		
		All patients	Non-anemic	Anemic
sTfR	ID	12 (22%)	4 (13%)	8 (35%)
	IR	30 (15%)	22 (13%)	8 (33%)
Relative risk (CI) for transfusion with ID		1.4^NS^ (0.8, 2.6)	1.0^NS^ (0.4﻿, 2.6)	1.0^NS^ (0.5, 2.3)
Ferritin	ID	24 (16%)	14 (12%)	10 (28%)
	IR	18 (18%)	12 (13%)	6 (55%)
Relative risk (CI) for transfusion with ID		0.9^NS^ (0.5﻿,1.6)	0.9^NS^ (0.5﻿,1.9)	0.5^NS^ (0.2﻿,1.1)

The relative risks and 95% confidence intervals (CI) for transfusion are given in the entire cohort as well as according to the presence of absence of anemia, using the two definitions of ID. Where the CI for the relative risk includes 1 the difference is marked as NS (non-significant)

## Postoperative hospital stay

Iron deficiency identified by sTfR was associated with prolongation of hospital LoS from a mean (SD) of 6.3 (3.2) days in the IR_sTfR_ group to 8.5 (10.4) days in the ID_sTfR_ group (mean increase 2.2 days; 95% CI: 0.5–3.9; *P* = 0.011). ID_sTfR_ patients also had a longer CICU LoS, a mean of 2.5 (8.6) days compared with 1.2 (0.9) days for IR_sTfR_ patients (mean increase 1.3 days; 95% CI: 0.1–2.5; *P* = 0.039) (Table 5). Importantly, this association was not present in anemic patients and was absent when a ferritin-based definition of ID was used.

**Table 5:** Associations of iron status and anemia with length of stay

		Length of hospital stay in days, mean (SD)			Length of cardiac intensive care unit stay in days, mean (SD)		
		All patients	Non-anemic	Anemic	All patients	Non-anemic	Anemic
sTfR	ID	8.5 (10.4)	8.5 (12.9)	7.0 (2.5)	2.5 (8.6)	3.1 (11.1)	1.1 (0.6)
	IR	6.3 (3.2)	6.2 (3.2)	8.4 (5.5)	1.2 (0.9)	1.3 (1.0)	1.7 (2.2)
Difference (95% CI)		2.2* (0.5﻿, 3.9)	2.4* (0.1﻿, 4.6)	1.4^NS^ (−1.1﻿, 3.9)	1.3* (0.1﻿, 2.5)	1.9* (0.2﻿, 3.6)	0.6^NS^ (−0.3﻿, 1.6)
Ferritin	ID	6.9 (6.6)	6.6 (7.2)	7.7 (4.6)	1.7 (5.2)	1.8 (6.0)	1.3 (1.6)
	IR	6.7(3.9)	6.5 (3.9)	7.9 (4.6)	1.3 (1.1)	1.2 (1.0)	1.6 (1.9)
Difference (95% CI)		0.2^NS^ (− 1.3﻿, 1.6)	0.1^NS^ (− 1.6﻿, 1.7)	− 0.2^NS^ (− 3.2﻿, 2.8)	0.4^NS^ (− 0.7, ﻿1.4)	0.5^NS^ (− 0.7﻿, 1.8)	−0.3^NS^ (−1.5﻿, 0.8)

Values are given as the mean (SD). For comparisons between groups the mean difference and 95% confidence interval (CI) are given. Where the CI includes zero the difference is marked as NS (non-significant); where the CI does not include zero an asterisk indicates a statistically significant difference

## Discussion

In our cohort of patients undergoing surgical AVR, we found the prevalence of ID to be 60% using a conventional ferritin-based definition but only 22% using sTfR. Our main finding is that ID predicted significantly longer hospital and CICU stays. Interestingly, this was only true when using sTfR to identify ID; ferritin did not have this discriminatory value. Furthermore, the association with prolonged LoS in ID_sTfR_ patients was unexpectedly confined to those who were not anemic, reinforcing that the absence of clinically significant ID cannot safely be inferred from the absence of anemia.

In a recent observational study of patients with severe aortic stenosis referred for AVR [24], the prevalence of ID using a ferritin and TSat-based definition was reported to be 53%, and the prevalence of anemia 20%, very similar figures to ours. Although sTfR was measured in that study, associations with LoS were not reported. Another study examined transcatheter aortic valve implantation [25] but iron indices were only assayed in anemic individuals, meaning that no conclusions can be drawn regarding NAID.

In a study that employed a complex algorithm for defining ID, including ferritin, TSat, CRP and sTfR, no association of preoperative ID with CICU LoS was seen, although iron-deficient patients did report significantly increased postoperative fatigue [14]. Conversely, a serum ferritin < 100 µg/L in isolation was associated with increased mortality, major cardiac and cerebrovascular events, transfusion, and prolonged hospital stay in one randomised controlled trial (RCT) [26].

That we were able to identify associations with sTfR but not ferritin in a smaller, more homogenous group might imply that sTfR is superior to ferritin at identifying clinically relevant ID, at least in part because sTfR is not influenced by inflammation [27]. Alternatively, it may be that patients undergoing surgical AVR differ from a mixed cardiac surgical population. None of our patients died, and none underwent simultaneous surgical revascularisation. Differing levels of subclinical inflammation in patients undergoing other cardiac surgical procedures–particularly coronary artery bypass grafting–may be important.

Our study has several limitations. First, it was not possible to measure TSat owing to the absence of serum samples. Second, we were limited to the outcome data originally collected in the MAVRIC trial. Third, because MAVRIC was a 270-patient single centre trial, the present work may have been underpowered to detect other clinically significant associations.

A major challenge when investigating the interplay between iron homeostasis and CVD is the manner in which ID is defined. Whilst the definition based on ferritin and TSat that has generally been adopted in CVD [5] certainly identifies a group in whom there is a net benefit from IV iron therapy [6], it must be noted that it is arbitrary and does not have a firm pathobiological basis. Consequently, this definition may miss a group of patients who would benefit from IV iron therapy, whilst simultaneously including some individuals who derive no benefit or may be harmed.

Our data suggest that the use of sTfR avoids misclassifying patients as iron replete who in fact have inflammation-driven elevation of ferritin [27], whilst simultaneously not falsely labelling as iron deficient those healthy individuals with a ferritin < 100 µg/L, which may still be consistent with healthy iron stores and biologically available iron [28]. In support of this possibility, levels of the master iron-regulatory hormone hepcidin were similar in our iron-deficient patients whether identified as such by a low ferritin or high sTfR. Hepcidin is ordinarily heavily suppressed by absolute ID, leading to increased iron uptake from the gut and its liberation from reticuloendothelial stores. Elevated levels of hepcidin seen with infection and inflammation reflect a mechanism to sequester iron and reduce its bioavailability to pathogens [29]. Patients in the present study identified as iron replete based on ferritin ≥ 100 µg/L had hepcidin levels ~ 50% higher than those identified as iron replete based on sTfR, arguing against any great additional diagnostic value of hepcidin measurement in this setting.

## Conclusion

We have found a novel association between NAID and prolonged hospital and CICU LoS in patients undergoing surgical AVR. Whilst it is not possible from the present study to determine the underlying mechanisms, our findings clearly identify elevated sTfR in non-anemic cardiac surgery patients as a treatable trait that might improve postoperative recovery and shorten hospital stay. They also support a future RCT of iron supplementation as a targeted therapy in this group, rather than an indiscriminate intervention aimed primarily at reducing perioperative blood transfusion requirements.

## Data Availability

The datasets used and/or analysed during the current study are available from the corresponding author on reasonable request.

## Abbreviations

AVR: Aortic valve replacement
CHF: Chronic heart failure
CICU: Cardiac intensive care unit
CRP: C-reactive protein
CVD: Cardiovascular disease
ID: Iron deficiency
IDA: Iron-deficiency anemia
IR: Iron repletion
IV: Intravenous
LoS: Length of stay
NAID: Non-anemic iron deficiency
NHS: National health service
RCT: Randomised controlled trial
SD: Standard deviation
sTfR: Soluble transferrin receptor
TSat: Transferrin saturation
